# Supplementary figures and images for: Plant growth promoting endophyte Burkholderia contaminans NZ antagonizes phytopathogen Macrophomina phaseolina through melanin synthesis and pyrrolnitrin inhibition
Source: PLoS One. 2021 Sep 30;16(9):e0257863. doi: 10.1371/journal.pone.0257863 (PMC8483353; doi:10.1371/journal.pone.0257863)

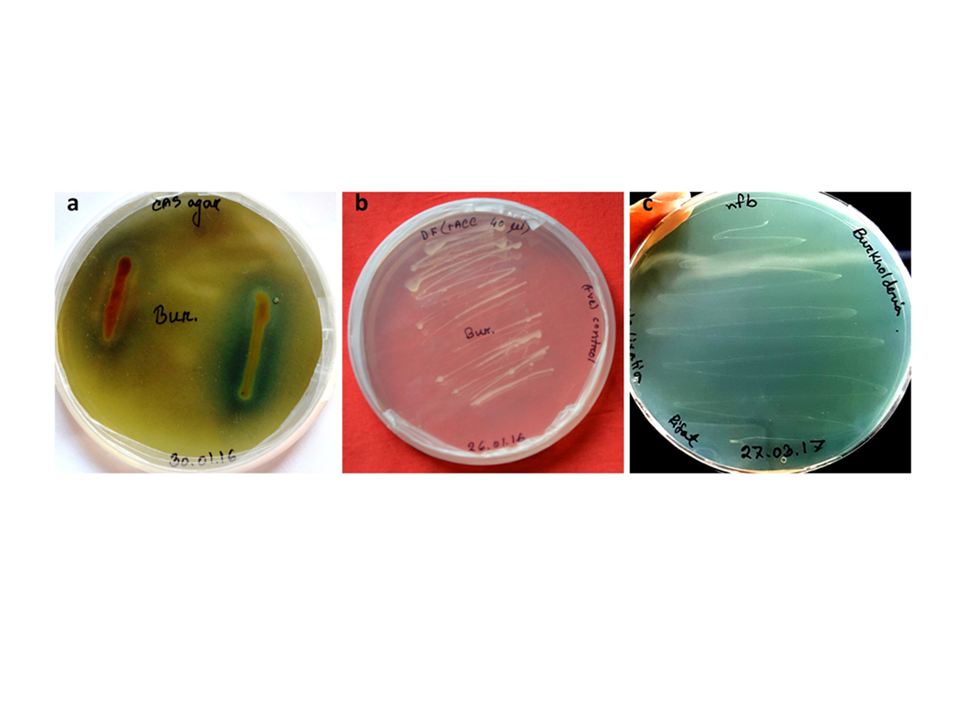

Supplement: S1 Fig — (TIF) [file pone.0257863.s001.tif]

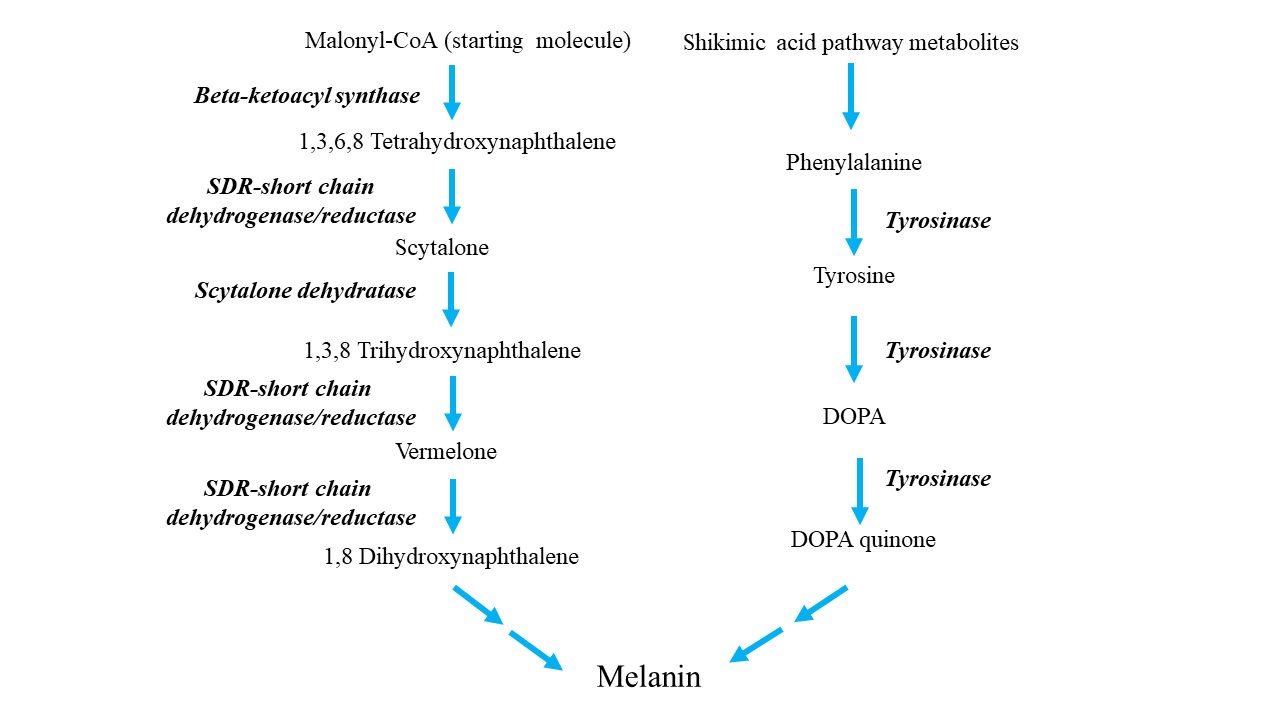

Supplement: S2 Fig — Qualitative assay for (a) siderophore, (b) ACC deaminase, and (c) nitrogen of B. contaminans NZ. (TIF) [file pone.0257863.s002.tif]
