# Supplementary material for: Plant growth promoting endophyte Burkholderia contaminans NZ antagonizes phytopathogen Macrophomina phaseolina through melanin synthesis and pyrrolnitrin inhibition
Source: PLoS One. 2021 Sep 30;16(9):e0257863. doi: 10.1371/journal.pone.0257863 (PMC8483353; doi:10.1371/journal.pone.0257863)
Supplement: S1 Table — The data of three replicates per experiment are presented as means and standard deviations. (DOCX) [file pone.0257863.s003.docx]

**S1 Table.** Average root length, shoot length and plant height of bacteria treated jute seedlings vs untreated control in pot experiment in 4, 7 and 10 days. Mean and standard deviation of three replicates per experiment are presented.

|  |  | Average Root Length (cm) | SD | SE | p value | Average Shoot Length (cm) | SD | SE | p value |
| --- | --- | --- | --- | --- | --- | --- | --- | --- | --- |
| Day 4 | Control | 1.64 | 0.11 | 0.05 | 0.0007 | 1.40 | 0.32 | 0.14 | 0.01114 |
|  | *Burkholderia* | 2.5 | 0.34 | 0.15 |  | 2.22 | 0.46 | 0.21 |  |
| Day 7 | Control | 4.06 | 0.52 | 0.23 | 0.0047 | 1.26 | 0.22 | 0.10 | 0.0083 |
|  | *Burkholderia* | 6.18 | 1.11 | 0.49 |  | 1.92 | 0.36 | 0.16 |  |
| Day 10 | Control | 5.2 | 0.95 | 0.42 | 0.000035 | 1.42 | 0.18 | 0.08 | 0.00001 |
|  | *Burkholderia* | 11.28 | 1.35 | 0.60 |  | 2.44 | 0.15 | 0.07 |  |
|  |  |  |  |  |  |  |  |  |  |

|  |  | Average Height (cm) | SD | SE |
| --- | --- | --- | --- | --- |
| Day 4 | Control | 3.04 | 0.42 | 0.19 |
|  | *Burkholderia* | 4.72 | 0.44 | 0.20 |
| Day 7 | Control | 5.32 | 0.68 | 0.31 |
|  | *Burkholderia* | 8.10 | 1.01 | 0.45 |
| Day 10 | Control | 6.62 | 1.04 | 0.46 |
|  | *Burkholderia* | 13.72 | 1.09 | 0.48 |
|  |  |  |  |  |
