# Supplementary material for: Plant growth promoting endophyte Burkholderia contaminans NZ antagonizes phytopathogen Macrophomina phaseolina through melanin synthesis and pyrrolnitrin inhibition
Source: PLoS One. 2021 Sep 30;16(9):e0257863. doi: 10.1371/journal.pone.0257863 (PMC8483353; doi:10.1371/journal.pone.0257863)
Supplement: S2 Table — The data of three replicates per experiment are presented as mean(s) and standard deviation(s). (DOCX) [file pone.0257863.s004.docx]

**S2 Table.** Fresh weight and dry weight of the bacteria treated jute seedlings vs untreated control in pot experiment in 4, 7 and 10 days with mean and standard deviation of three replicates.

|  | | **Average Fresh wt (mg)** | **p value** | **SD** | **SE** | **Average Dry wt (mg)** | **SD** | **SE** | **p value** |
| --- | --- | --- | --- | --- | --- | --- | --- | --- | --- |
| **Day 4** | Control | 8.98 | 0.02 | 2.44 | 1.09 | 1.66 | 0.43 | 0.19 | 0.04 |
|  | *Burkholderia* | 21.64 |  | 1.60 | 0.69 | 2.40 | 0.50 | 0.23 |  |
| **Day 7** | Control | 21.64 | 0.00043 | 1.69 | 0.75 | 5.20 | 0.75 | 0.33 | 0.00002 |
|  | *Burkholderia* | 47.96 |  | 10.08 | 4.51 | 8.80 | 0.52 | 0.23 |  |
| **Day 10** | Control | 87.98 | 0.00051 | 8.82 | 3.94 | 15.68 | 1.32 | 0.59 | 0.00003 |
|  | *Burkholderia* | 123.86 |  | 11.27 | 5.04 | 21.26 | 0.69 | 0.31 |  |
