# Supplementary material for: Plant growth promoting endophyte Burkholderia contaminans NZ antagonizes phytopathogen Macrophomina phaseolina through melanin synthesis and pyrrolnitrin inhibition
Source: PLoS One. 2021 Sep 30;16(9):e0257863. doi: 10.1371/journal.pone.0257863 (PMC8483353; doi:10.1371/journal.pone.0257863)
Supplement: S3 Table — (DOCX) [file pone.0257863.s005.docx]

**S3 Table.** Genomic features of *Burkholderia contaminans* NZ

| **Genomic features of *Burkholderia contaminans* NZ** | **Genome characteristics** |
| --- | --- |
| Genome size (bps) | 8742352 |
| No. of contigs | 2211 |
| No. of scaffolds | 2099 |
| N50 | 6571 |
| L50 | 402 |
| GC content (%) | 66.6% |
| No. of protein coding genes | 7805 |
| No. of tRNA coding genes | 76 |
| NCBI accession no. | QRBC00000000 |
