# Supplementary material for: Plant growth promoting endophyte Burkholderia contaminans NZ antagonizes phytopathogen Macrophomina phaseolina through melanin synthesis and pyrrolnitrin inhibition
Source: PLoS One. 2021 Sep 30;16(9):e0257863. doi: 10.1371/journal.pone.0257863 (PMC8483353; doi:10.1371/journal.pone.0257863)
Supplement: S4 Table — (DOCX) [file pone.0257863.s006.docx]

| **Properties** | **Name** | **Biosynthetic genes** | **Seq location** | **start** | **stop** |
| --- | --- | --- | --- | --- | --- |
| Phytohormone production and stress alleviation | Management of ethylene stress | ACC (1-Aminocyclopropane-1-carboxylic acid) deaminase | Seq562_length_5214_cov_3.44879 | 1445 | 2461 |
|  | Production of IAA (Indole Acetic Acid) | Indole-3-glycerate phosphate synthase | Seq802_length_3768_cov_2.91733 | 582 | 1367 |
|  |  | Indole pyruvate oxidoreductase | Seq1081_length_2581_cov_3.50244 | 151 | 1497 |
|  |  | Indole acetamide hydroxylate | Seq1046_length_2690_cov_2.33632 | 179 | 763 |
|  |  | Tryptophan synthase | Seq274_length_8346_cov_3.55031 | 4434 | 5627 |
|  |  | Nitrile hydratase subunit alpha | Seq393_length_6908_cov_3.57912 | 5597 | 6196 |
|  |  | Nitrile hydratase subunit beta | Seq393_length_6908_cov_3.57912 | 6241 | 6783 |
| Phosphate solubilization | pyrroloquinoline quinone gene | *pqq* | Seq387_length_6923_cov_4.05518 | 2151 | 3872 |
|  | Enolase | *eno* | Seq1553_length_1281_cov_3.32236 | 815 | 93 |
| Nitrogen fixation | nif gene cluster | *nif HDK, nifQ,* | Seq1101_length_2501_cov_3.72199 | 1723 | 1124 |
|  | Others | *nodT, nir, nor, nolO* | Seq1827_length_697_cov_2.6386 | 261 | 611 |
| Antibiotic biosynthesis | Pyrrolnitrin | *prnA-prnD* | Seq236_length_8953_cov_4.2291 | 568 | 5049 |
| Siderophore Biosynthesis | Polychelin | *pchR* | Seq747_length_4108_cov_2.41145 | 1482 | 541 |
|  | Ferric siderophore transport | *pchD-pchA* | Seq522_length_5532_cov_4.02239_59_793 | 59 | 793 |
|  | ABC-type siderophore export system | *feoB* | Seq541_length_5390_cov_3.94148_1970_3673 | 1970 | 3673 |
|  | Siderophore pyoverdine | *pvd* | Seq1988_length_485_cov_0.849162 | 457 | 104 |

**S4 Table.** Location of genes involved in plant growth promotion activity detected from RAST, antiSMASH, and PIFAR analysis of the whole genome of *B. contaminans* NZ.
