# Supplementary material for: Plant growth promoting endophyte Burkholderia contaminans NZ antagonizes phytopathogen Macrophomina phaseolina through melanin synthesis and pyrrolnitrin inhibition
Source: PLoS One. 2021 Sep 30;16(9):e0257863. doi: 10.1371/journal.pone.0257863 (PMC8483353; doi:10.1371/journal.pone.0257863)
Supplement: S5 Table — (DOCX) [file pone.0257863.s007.docx]

**S5 Table:** *In vitro* plant growth promotion attributes of *B. contaminans* NZ

| Lists of assays | Observations | Remarks |
| --- | --- | --- |
| ACC deaminase activity | 1. Bacterial growth in DF minimal salt medium in plate assay (supplementary Fig 2b). 2. Production of 54.2 µM mg^-1^ h^-1^ of α-ketobutyrate by hydrolyzing ACC in quantitative assay. | **+** |
| *In vitro* production of IAA | Addition of Salkowski's reagent in the bacterial culture supernatant produced 12.22 μg/ml IAA | **+** |
| Siderophore production | Yellow halo of 6 mm formed around the bacterial colony on a CAS plate (supplementary Fig. 2a) | **+** |
| Biological nitrogen fixation | Bacterial growth observed in Nfb solid and semisolid media (supplementary Fig. 2c) | **+** |

**(**+ sign indicates presence of the tested feature responsible for plant growth promotion**)**
