# Supplementary material for: Plant growth promoting endophyte Burkholderia contaminans NZ antagonizes phytopathogen Macrophomina phaseolina through melanin synthesis and pyrrolnitrin inhibition
Source: PLoS One. 2021 Sep 30;16(9):e0257863. doi: 10.1371/journal.pone.0257863 (PMC8483353; doi:10.1371/journal.pone.0257863)
Supplement: Attachment — Submitted filename: APAP.docx [file pone.0257863.s008.docx]

Dear Editor and Authors,

On the basis of what the authors reported in the manuscript, the present study was aimed to "Plant growth promoting endophyte Burkholderia contaminants NZ antagonizes phytopathogen *Macrophomina phaseolina* through multiple modes of action".

To gain these aims, the authors developed series of experiments devoted to re-culturing, identify pathogen and growth promoting bacterial isolates using GC-MS and whole genome sequencing and the information in results is clear and concise but need to be checked again as there are few grammatical error and used a few long sentences that should be summarized.

Materials and methods should be more attractive if the sequencing and analysis be placed before the *in-vivo* and *in-vitro* studies; over all the manuscript is written well and have valuable information for the readers. The whole manuscript should be proofread for English language editing and grammatical errors.

Discussion section of the manuscript is should be improved by using a few latest references.
