# Supplementary material for: Plant growth promoting endophyte Burkholderia contaminans NZ antagonizes phytopathogen Macrophomina phaseolina through melanin synthesis and pyrrolnitrin inhibition
Source: PLoS One. 2021 Sep 30;16(9):e0257863. doi: 10.1371/journal.pone.0257863 (PMC8483353; doi:10.1371/journal.pone.0257863)
Supplement: Attachment — Submitted filename: Responses to the reviewers comment.docx [file pone.0257863.s009.docx]

**Responses to the Editor and Reviewers’ comments**

1. Please ensure that your manuscript meets PLOS ONE's style requirements, including those for file naming. The PLOS ONE style templates can be found at
   <https://journals.plos.org/plosone/s/file?id=wjVg/PLOSOne_formatting_sample_main_body.pdf> and 
   <https://journals.plos.org/plosone/s/file?id=ba62/PLOSOne_formatting_sample_title_authors_affiliations.pdf>

*The format has been changed according to PLOS ONE’s style***.**

2. Thank you for stating the following in the Acknowledgments Section of your manuscript:

We would like to address the funding information as stated below:

*This project is funded by Higher Education Quality Enhancement Project (HEQEP) (Grant number: CP-3250), a World Bank financed development project. The funders had no role in study design, data collection and analysis, decision to publish, or preparation of the manuscript."*

3. In your Data Availability statement, you have not specified where the minimal data set underlying the results described in your manuscript can be found. PLOS defines a study's minimal data set as the underlying data used to reach the conclusions drawn in the manuscript and any additional data required to replicate the reported study findings in their entirety. All PLOS journals require that the minimal data set be made fully available. For more information about our data policy, please see <http://journals.plos.org/plosone/s/data-availability>.

"Upon re-submitting your revised manuscript, please upload your study’s minimal underlying data set as either Supporting Information files or to a stable, public repository and include the relevant URLs, DOIs, or accession numbers within your revised cover letter. For a list of acceptable repositories, please see <http://journals.plos.org/plosone/s/data-availability#loc-recommended-repositories>. Any potentially identifying patient information must be fully anonymized.

Important: If there are ethical or legal restrictions to sharing your data publicly, please explain these restrictions in detail. Please see our guidelines for more information on what we consider unacceptable restrictions to publicly sharing data: <http://journals.plos.org/plosone/s/data-availability#loc-unacceptable-data-access-restrictions>. Note that it is not acceptable for the authors to be the sole named individuals responsible for ensuring data access.

We will update your Data Availability statement to reflect the information you provide in your cover letter.

*There are no ethical or legal restrictions to sharing our data publicly. Data Availability statement has been corrected to ‘The authors have no objection to make the data set underlying the results described in our manuscript to be fully available.’*

*In the revised cover letter the accession no of the bacterial whole genome sequence (This Whole Genome Shotgun project has been deposited at DDBJ/ENA/GenBank under the accession QRBC00000000) and data availability statement have been included.*

4. PLOS requires an ORCID iD for the corresponding author in Editorial Manager on papers submitted after December 6th, 2016. Please ensure that you have an ORCID iD and that it is validated in Editorial Manager. To do this, go to ‘Update my Information’ (in the upper left-hand corner of the main menu), and click on the Fetch/Validate link next to the ORCID field. This will take you to the ORCID site and allow you to create a new iD or authenticate a pre-existing iD in Editorial Manager. Please see the following video for instructions on linking an ORCID iD to your Editorial Manager account: <https://www.youtube.com/watch?v=_xcclfuvtxQ>

*An ORCID iD was generated and validated in Editorial Manager*

5. We note that you have included the phrase “**data not shown**” in your manuscript. Unfortunately, this does not meet our data sharing requirements. PLOS does not permit references to inaccessible data. We require that authors provide all relevant data within the paper, Supporting Information files, or in an acceptable, public repository. Please add a citation to support this phrase or upload the data that corresponds with these findings to a stable repository (such as Figshare or Dryad) and provide and URLs, DOIs, or accession numbers that may be used to access these data. Or, if the data are not a core part of the research being presented in your study, we ask that you remove the phrase that refers to these data.

*The results have been included in the Supporting Information (S2 Fig and S5 Table)*

6. We note that Figures 1, 2, and 3 in your submission contain copyrighted images. All PLOS content is published under the Creative Commons Attribution License (CC BY 4.0), which means that the manuscript, images, and Supporting Information files will be freely available online, and any third party is permitted to access, download, copy, distribute, and use these materials in any way, even commercially, with proper attribution. For more information, see our copyright guidelines: <http://journals.plos.org/plosone/s/licenses-and-copyright>.
 
*There is no chance of copyright issue for Figures 1, 2 and 3. They have been produced in this study and have not been used in any other article or report.*

Additional Editor Comments (if provided):

The English language must be addressed with the help of a native speaker or English language editing service. Furthermore, the manuscript lacks detailed experimental procedure; at least three replications and one repetition of experiment are required, failing which MS will be rejected. Discussion must focus the critical analysis of literature and should be condensed. Conclusion summarizes the findings of research.

[Note: HTML markup is below. Please do not edit.]

**Reviewers' comments:**

Reviewer's Responses to Questions

**Comments to the Author**

1. Is the manuscript technically sound, and do the data support the conclusions?

The manuscript must describe a technically sound piece of scientific research with data that supports the conclusions. Experiments must have been conducted rigorously, with appropriate controls, replication, and sample sizes. The conclusions must be drawn appropriately based on the data presented.

Reviewer #1: Yes

Reviewer #2: Yes

2. Has the statistical analysis been performed appropriately and rigorously?

Reviewer #1: Yes

Reviewer #2: Yes

3. Have the authors made all data underlying the findings in their manuscript fully available?

The [PLOS Data policy](http://www.plosone.org/static/policies.action#sharing) requires authors to make all data underlying the findings described in their manuscript fully available without restriction, with rare exception (please refer to the Data Availability Statement in the manuscript PDF file). The data should be provided as part of the manuscript or its supporting information, or deposited to a public repository. For example, in addition to summary statistics, the data points behind means, medians and variance measures should be available. If there are restrictions on publicly sharing data—e.g. participant privacy or use of data from a third party—those must be specified.

Reviewer #1: Yes

Reviewer #2: Yes

4. Is the manuscript presented in an intelligible fashion and written in standard English?

PLOS ONE does not copyedit accepted manuscripts, so the language in submitted articles must be clear, correct, and unambiguous. Any typographical or grammatical errors should be corrected at revision, so please note any specific errors here.

Reviewer #1: Yes

Reviewer #2: Yes

5. Review Comments to the Author

Please use the space provided to explain your answers to the questions above. You may also include additional comments for the author, including concerns about dual publication, research ethics, or publication ethics. (Please upload your review as an attachment if it exceeds 20,000 characters)

**Reviewer #1:**

On the basis of what the authors reported in the manuscript, the present study was aimed to "Plant growth promoting endophyte Burkholderia contaminants NZ antagonizes phytopathogen Macrophomina phaseolina through multiple modes of action".
To gain these aims, the authors developed series of experiments devoted to re-culturing, identify pathogen and growth promoting bacterial isolates using GC-MS and whole genome sequencing and the information in results is clear and concise but need to be checked again as there are few grammatical error and used a few long sentences that should be summarized.
Materials and methods should be more attractive if the sequencing and analysis be placed before the in-vivo and in-vitro studies; over all the manuscript is written well and have valuable information for the readers. The whole manuscript should be proofread for English language editing and grammatical errors.
Discussion section of the manuscript is should be improved by using a few latest references.

*Changes has been made according to the suggestions.*

**Reviewer #2:**

1. Objective of the study should be more clearly defined.

*The objective has been clearly defined.*

2. Replicates used in the experiments are very few, so my question is why the authors did not repeat the experiments. If they did, then it requires proper elaboration and How will authors justify the findings and their reproducibility?

*All the experiments were done with three replications as stated in the Materials and Method section (lines 119 and 172) and all tests were performed in triplicate if not mentioned otherwise.*

3. Discussion is very long, it should be précised.

*Discussion has been modified accordingly.*

4. Conclusion section is lacking impressiveness in its expression. I will suggest authors to put an extra effort in improving it.

*This has been modified accordingly.*

5. There is also an suggestion about the title, I think it should be more specific as it is very generalized in its current form.

*The title has been edited as suggested.*

6. PLOS authors have the option to publish the peer review history of their article ([what does this mean?](https://journals.plos.org/plosone/s/editorial-and-peer-review-process#loc-peer-review-history)). If published, this will include your full peer review and any attached files.


If you choose “no”, your identity will remain anonymous but your review may still be made public.


**Do you want your identity to be public for this peer review?** For information about this choice, including consent withdrawal, please see our [Privacy Policy](https://www.plos.org/privacy-policy).

Reviewer #1: **Yes:** Raees Ahmed

Reviewer #2: No

**Reviewer Recommendation Term:**   

Major Revision

1. Is the manuscript technically sound, and do the data support the conclusions?       
Yes

2. Has the statistical analysis been performed appropriately and rigorously?   
Yes

3. Have the authors made all data underlying the findings in their manuscript fully available?
Yes

4. Is the manuscript presented in an intelligible fashion and written in standard English?
Yes

6. **Review Comments to the Author**

The data itself is good, but the manuscript need to be improved in terms of writing. There are so many small mistakes that can easily be fixed by just reading it through a few more times. Here I listed a portion of the problems, but I recommend the authors to go over carefully for each sentence and craft it again. Also, I didn't like the red background for some figures. Although I won't say this is mandatory, I think red is probably not the best background color for those figures.
Overall, this paper presents solid data sets and I recommend it to be published after major revisions.

**Abstract**
Line 40- Secondary metabolites, catechols and ergotaman (that has been found through what?)

*They were found through GC-MS analysis as mentioned in line 35.*

**Introduction**

Line 58- be specific of what 'They' indicates (ex: the beneficial effects of endophytes)

*Revised accordingly*

Line 61-63
biocontrol activity including competition for iron, nutrient and space, production of antibiotics, lytic enzymes and volatile compounds, and induction of systemic resistance.

*Corrected accordingly*
Line 63-65
Many studies have emphasized the ability of these microorganisms for possible roles for promoting plant growth and protection through additive/synergistic effects.

*Corrected accordingly*
Line 66
B. subtilis [9], Pseudomonas parafulva, and Pantoea agglomerans are a few examples.
Not so sure about "recent developments". What developments?

*Corrected accordingly*
Line 68
Full scientific name for Jute
Throughout its life cycle,

*Corrected accordingly*
Line 81
human environment? Like inside of human body? If so,
"a widespread presence in water, soil, plants, and animals including human"

*Corrected accordingly*
Line 84 & 86
Space between (Bcc) and [17]        /    [18] and .

*Corrected accordingly*
Line 90
Don't start a line with "but"

*Corrected accordingly*
Line 94-96
I think this line can be written shorter and clearer.

*Changed accordingly*
Overall, introduction is good, but can be better by reorganizing and rewriting sentences.
Line 105
Delete "used in this study ... collection of"

*Corrected accordingly*

**Materials and methods**
Line 108-109 and throughout manuscript
Use full scientific names including authroties if possible.
*These should be okay.*

Line 117
Fix 'NZ' (italic)

*Corrected accordingly*
Line 123
At first,
TSB media? Full names should be mentioned when first introduced. Instead of that, full name was introduced in line 199

*Corrected accordingly*
Line 124
At the same time,

*Corrected accordingly*
Line 134
and MgSO4.7H2O

*Corrected accordingly*
Line 137
Thirty instead of 30

*Corrected accordingly*
Line 152
(10x coverage)

*Corrected accordingly*
Line 158
server [29]

*Corrected accordingly*
Line 198 and throughout manuscript
Authors should be consistant for number+unit (ul, degree, etc) combination. For example, I can see inconsistancies such as 20ml (no space) vs 20 ml (space between number+unit). Just go with one.

*Corrected accordingly*
Line 205-206
For HPLC and LC-MS analysis, a seperate 1000ml of B. contaminans NZ was cultured under similar conditions described above.

*Corrected accordingly*
Line 208
Delete 'together' since co-culture already contains the meaning.

*Corrected accordingly*
Line 209
filtered thorough 'what'?

*Corrected accordingly*
Line 213-215
Recommend to rephrase it. Hard to understand.

*Revised accordingly*
Line 220
One µL is awkward. What about one microliter?

*Corrected accordingly*

Line 221
Helium was used as the carrier gas with the flow rate set at 1.0 mL/min
Also, be comsistant with ml or mL (chose one)

*Corrected accordingly*
Line 224
respectively, and

*Corrected accordingly*
Line 235
temperature:

*Corrected accordingly*
Line 251-252
The capillary voltage was maintained at 4000 V, and dry gas temperature was set at 350°C.

*Corrected accordingly*
Line 256
biosynthesis; among those,

*Corrected accordingly*

**Results**
Table 1.
Adding chromosomal locations for the genes would be beneficial.

*Has been added in S4 Table*
Line 334
and (f) Rhizoctonia solani

*Corrected accordingly*
Table 3.
Xylaria sp. 
Nigrospora sphaerica 52.99 ± 3.67

*Corrected accordingly*
Line 403-404
, and their 404 corresponding homologues in M. phaseolina were identified through a BLAST search

*Corrected accordingly*

**Discussion**
Line 416
Delete ,

*Corrected accordingly*
Line 440
Delete But and replace with 'however' or similar word.

*Corrected accordingly*

Line 458
The Burkholderia genome

*Corrected accordingly*
Line 459
M. phaseolina

*Corrected accordingly*
Line 461
and Rhizoctonia solani

*Corrected accordingly*
Line 481
Space issue

*Corrected accordingly*
Line 530
, and scytalone dehydratase

*Corrected accordingly*
Line 547
downregulation

*Corrected accordingly*
