# Supplementary material for: Plant growth promoting endophyte Burkholderia contaminans NZ antagonizes phytopathogen Macrophomina phaseolina through melanin synthesis and pyrrolnitrin inhibition
Source: PLoS One. 2021 Sep 30;16(9):e0257863. doi: 10.1371/journal.pone.0257863 (PMC8483353; doi:10.1371/journal.pone.0257863)
Supplement: Attachment — Submitted filename: Response to the Editors Comments.docx [file pone.0257863.s010.docx]

**Responses to the Editor and Reviewers’ comments**

**Additional Editor Comments (if provided):**

This manuscript is an improvement over the previously submitted version. Although all reviewers recommended publication of the MS, however, The MS still needs improvement in terms of clarity and quality. I recommended in my last review that the authors find someone fluent in scientific writing in English to correct the many grammar, terminology, and sentence structure errors in the manuscript. While, some effort was made to correct these errors, the current manuscript is still filled with errors that make the article very difficult to read. I have noted a few of these errors on the manuscript, but there are many errors per page that need to be addressed. The level of writing is not up to the standard required for publication in PLoS series journals. At this point, the authors should consider employing a professional editor experienced in scientific writing.

*The revised manuscript has been corrected by professional editing company.*

In abstract, background of research and aim of the study are missing, please revise abstract to make it more focused.

*Made changes according to the suggestions*

What is NZ in abstract, this is a wrong way of presentation please revise, do you mean Burkholderia contaminans, if it is, then it is not properly cited.

*Changed all instances of NZ to B. contaminans NZ*

Page 2 lines 34 compounds or molecules not substances, and overall the MS need thorough English revision failing which MS will be not accepted.

*Changed according to the suggestions*

Overall, abstract is very poorly written, please revise it to make more focused.

*Revised according to the suggestions*

Page 3 line 65 line 85 species not italic correct in whole MS

*Revised accordingly*

Line 77 space

*Revised accordingly*

Line 92 to 101 it should described objective of your research and why it needed and then what are you presenting in this paper.

*Revised accordingly*

Did you check pathogenicity of *Macrophomina phaseolina* before conducting experiments?

*We have checked the pathogenicity of Macrophomina phaseolina on jute seedlings and found jute seedlings dies after few days of infection, and turns brown. This result has been included in our recent paper by Zaman NR et al. (2020)*

# Ref:

# 1. Zaman NR, Kumar B, Nasrin Z, Islam MR, Maiti TK, Khan H. (2020) Proteome Analyses Reveal *Macrophomina phaseolina*'s Survival Tools When Challenged by *Burkholderia contaminans* NZ. *ACS Omega*. 5(3):1352-1362. doi:10.1021/acsomega.9b01870

Line 120 it was not grown in shaker, you incubated in incubator shaker, how long old culture you used for DNA isolation

*The statement was corrected according to the suggestion. It is mentioned in the same line that the bacterium was grown overnight and DNA was isolated from that culture.*

Line 172, the experiment was repeated three times, if it is the case, how you handled the data from repeated experiments it is not described in the statistical section. Also this section does not show you used original data or transformed data for analysis.

*The average shoot length, average root length and average height from the three repeated experiment was taken for statistical analysis shown in supplementary table 1.*

Petri dish, P should be capital

*Revised accordingly*

Remove statistical part from each section and put it in the last of material and method in the single section describing the details how you analyzed the data as I mentioned.

*Revised accordingly*

Line 259 ascomycota group of fungi
*Revised accordingly*

Line 272 (P<0.05)

*Revised accordingly throughout the MS*

Table1 I think name of genes should be italic, follow in all tables

*Revised accordingly*

Coulumn one Fungal Species

*Revised accordingly*

Line 342 full name *Macrophomina phaseolina* should start follow in whole in whole MS same trend

*Revised accordingly*

Table 4 can be given as supplementary table

*Revised accordingly*

Line 441 strains

*Revised accordingly*

Conclusion should be short and easily understandable

*Changed accordingly*

Some of the references not follow the journal trends.

*Changed accordingly*
